# Making space for empathy: supporting doctors in the emotional labour of clinical care

**DOI:** 10.1186/s12910-016-0091-7

**Published:** 2016-01-27

**Authors:** Angeliki Kerasidou, Ruth Horn

**Affiliations:** Researcher in Global Health Ethics, The Ethox Centre, Nuffield Department of Population Health, University of Oxford, Old Road Campus, Oxford, OX3 7LF UK; Ethics and Society Wellcome Trust Fellow & Reseacher in Ethics, The Ethox Centre, Nuffield Department of Population Health, University of Oxford, Oxford, UK; The Ethox Centre, Nuffield Department of Population Health, University of Oxford, Old Road Campus, Oxford, OX3 7LF UK

**Keywords:** Empathy, Emotional detachment, Emotional over-involvement, Professionalism

## Abstract

**Background:**

The academic and medical literature highlights the positive effects of empathy for patient care. Yet, very little attention has been given to the impact of the requirement for empathy on the physicians themselves and on their emotional wellbeing.

**Discussion:**

The medical profession requires doctors to be both clinically competent and empathetic towards the patients. In practice, accommodating both requirements can be difficult for physicians. The image of the technically skilful, rational, and emotionally detached doctor dominates the profession, and inhibits physicians from engaging emotionally with their patients and their own feelings, which forms the basis for empathy. This inhibition has a negative impact not only on the patients but also on the physicians. The expression of emotions in medical practice is perceived as unprofessional and many doctors learn to supress and ignore their feelings. When facing stressful situations, these physicians are more likely to suffer from depression and burnout than those who engage with and reflect on their feelings. Physicians should be supported in their emotional work, which will help them develop empathy. Methods could include questionnaires that aid self-reflection, and discussion groups with peers and supervisors on emotional experiences. Yet, in order for these methods to work, the negative image associated with the expression of emotions should be questioned. Also, the work conditions of physicians should improve to allow them to make use of these tools.

**Summary:**

Empathy should not only be expected from doctors but should be actively promoted, assisted and cultivated in the medical profession.

## Background

Contemporary bioethics and medical literature highlights the positive effects of empathy for patient care [1–3]. Very little attention, however, has been given to the impact of the requirement for empathy on the physicians themselves.^1^ Doctors are expected to be empathetic towards their patients, but the effect of this requirement on their emotional wellbeing is rarely acknowledged. In order to address this gap, this paper discusses the ways in which the emotional work that is necessary for empathy affects physicians themselves and their ability to cope with distressing situations.

Medical professionalism puts the patients’ wellbeing at the fore; yet, doctors’ needs ought to be considered too. Empathy requires a level of emotional involvement from the side of the physician but the emotional resources required and the skills necessary for empathy are not always available to doctors. The medical profession is an emotionally challenging environment, which favours the image of the emotionally detached doctor. Often, in practice, the open expression of feelings is perceived as weakness, an attitude that leaves little room for the active pursuit of emotional wellbeing [4, 5]. Physicians are constantly faced with distressing situations, which can lead to depression, burnout, and subsequently loss of the ability to empathise [6, 7]. In order for empathy to flourish in the medical profession, doctors should feel able to deal with their emotions without the fear of being criticised or stigmatised as weak.

In this paper we argue that a shift is necessary in the medical profession that would create space for physicians to acknowledge and reflect on their feelings and would free resources to support them in their emotional work.

## Discussion

### Tensions in medical professionalism

Medical professionalism defines the types of skills and knowledge required by physicians and articulates their professional values, duties and obligations. It helps physicians navigate through difficult decisions required by their profession and offers them a guide of good practice. In other words, professionalism can be described as physicians’ practical and “moral compass” [8]. Two basic values for doctors are clinical competency and empathy. Both values are important elements of good medical practice; however, in many Western countries, there is a tendency to favour the technically skilful, rational, and emotionally detached physician rather than that of the compassionate or empathetic doctor. The active expression of compassion in medical care is often seen as a supererogatory requirement [9] or even perceived as weakness [4, 5]. As Coulehan and Williams note, doctors from early on in their training are taught that “technical skills are [considered] fundamental, whereas interactive skills (if encouraged at all) are secondary” [5].

There are several arguments in favour of emotional detachment of physicians. Lief and Fox [10] demonstrated how physicians learn during their studies to develop emotional detachment in order to maintain scientific and medical objectivity when dealing with distressing situations. Conversely, emotional attachment to patients is often seen as adverse to good clinical practice. An example of this are the General Medical Council’s guidelines [11], advising doctors against treating members of their families, or others with whom they have a close personal relationship. Strong emotional involvement and over-identification with patients has been linked with a tendency to over-treat without considering the side-effects [12]. Furthermore, emotional detachment allows physicians to remain composed when faced with emotionally difficult situations, and guide and support the patient through it. A doctor who crumbles and cries in front of a patient cannot fulfil this role. It puts the patient in the awkward position where they might feel obliged to support and comfort the physician, rather than the other way around. Physicians also need to retain an emotional detachment to protect themselves from stressful situations they face in their daily work [13]. Inability to manage emotions in medical practice is often perceived as lack of professionalism [14, 15].

However, protecting oneself from emotional distress by *overly* disconnecting from other persons, what can be described as apathy, can put good medical care at risk. Maslach maintains that “with increasing detachment comes an attitude of cold indifference to others’ needs and a callous disregard for their feelings”, which can result in the depersonalisation of the patient [16]. The NHS in recent years has placed a lot of emphasis on compassionate care in various guidelines and recommendations [17–19]. These recommendations underline the importance of empathy and compassion as fundamental requirements of professionalism in healthcare, when stating: “Most important of all, the NHS could employ hundreds of thousands of staff with the right technological skills, but without the compassion to care, then we will have failed to meet the needs of patients.” [20]. Yet, in practice it is difficult for physicians to find the right balance between being the technically skilled and objective professional, while being emotionally engaged, but not over-identifying with a patient’s distress. How can a professional maintain emotional distance while at the same time trying to understand the emotional life of their patients?

### Empathy as the middle way between emotional over- and under involvement

In the field of medical professionalism, empathy is presented as the ideal balance between emotional over-involvement and detachment. A number of different definitions of empathy have been proposed in the literature on how this balance should be understood and achieved in the medical practice [1, 21–23].

Empathy is often described as a process that engages both the cognitive and emotional domains of the person that empathises. The cognitive aspect of empathy refers to the ability an individual has to comprehend another person’s inner experience, and the capacity to understand the world from the other person’s stand point. The emotional aspect of empathy refers to the ability to join in someone else’s experiences and feelings [24, 25]. Compassion is the empathy-driven desire to act in order to address someone else’s needs and feelings [26, 27]. Joining into another person’s feelings, however, is not the same as sharing someone else’s feelings that is sympathy. According to Switankowsky [28] sympathy is the emotional identification with another individual, which can lead to emotional over-involvement, whereas empathy refers to the understanding of another person’s situation. Empathy, therefore, requires an emotional involvement with the other without, however, assuming their emotional position or projecting own emotions onto them.

Empathy entails the ability to be attentive to the difference between own and others’ feelings. The empathetic and self-aware physician can remain emotionally detached and, at the same time, engage with their patients’ situation. The ability to empathise thus reduces the risk of the physician being immersed into the patient’s suffering [21]. A number of studies have demonstrated the importance of empathy for patient care. It is associated with increased patient satisfaction, improved adherence to therapy, decreased medical errors, fewer malpractice claims, and better outcomes [29, 30]. However, little attention has been paid to the effects empathy or compassion has on physicians’ emotional wellbeing. In practice, it is difficult to strike the right balance between involvement and detachment. Rather than being pulled towards the one or the other extreme, physicians may choose to shy away from engaging emotionally with their patients [16, 22]. They find refuge in the image of the cool-minded medical professional and start thinking of empathy as an unnecessary or even irrational feeling.

Even when physicians consciously try to refrain from engaging emotionally with their patients, few will remain unaffected by the regular exposure to patients’ suffering, illness and death. Neglecting such emotions can seriously impact on the health and psychological wellbeing of these professionals. Physicians may suffer from emotional exhaustion, burnout, depression or even attempt suicide [6]. A recent study that included 14,000 doctors in Australia found that medical professionals are more likely to suffer from mental disorders and depression, than the general population; one of the main reasons being that ‘doctors and medical students are under immense pressure and deal regularly with pain and death’ [7]. Another study examining how distress and well-being relates to empathy showed that increasing personal and professional distress and emotional exhaustion are correlated with lower emotive empathy scores [31]. The authors suggest that efforts to promote empathy must consider these correlations.

Not engaging with emotions – one’s own emotions but also engaging emotionally with the other –has two adverse effects; firstly, it negatively affects doctors as persons, by increasing their stress and anxiety, making them more prone to mental suffering and emotional burnout. Secondly, it affects doctors as professionals, as it does not allow them to care for their patients’ appropriately and effectively. These two aspects of the doctor are integrally connected. If we are concerned with promoting best practice in medical care, then paying much more attention to the doctor as a person, and tending to their emotional and psychological wellbeing should become a priority too.

### Implementing empathy in practice

According to Davis, empathy develops through experience and by increasing self-awareness of one’s identity and personal values and boundaries [22]. The experiential aspect of empathy means that, as a professional skill, it cannot be directly taught. Its development, however, can be facilitated by creating the right conditions and providing the necessary tools and resources. Implementing such conditions and tools could help physicians properly integrate empathy into their professional practice and relationship with patients. In the following, we discuss possible steps that can be taken to facilitate this process.

As argued above, there is a tension in medical professionalism between the image of the clinically competent doctor and the caring doctor. Although the importance of compassion and empathy in healthcare is emphasised, it still remains the case that clinical competency is seen as the primary skill of medical practice. The ideal of the technically skilled doctor forms a big part of the medical culture and is incorporated in the medical training from early stages, whereas interpersonal and empathetic skills are seen as supererogatory and secondary [5].

The expression of emotions amongst physicians is often perceived as a sign of weakness and incompetence [32]. The stigma attached to admitting to psychological and emotional difficulties deters doctors from seeking help [33]. The ‘conspiracy of silence’, which often forms between the suffering doctor and their colleagues, means that the doctor is left alone to deal with difficult situations [32]. As a consequence, physicians appear to have higher chances of developing depression and other forms of mental illness than their peers [34–36]. Considering the negative effects of physicians suppressing their feelings and denying their need for support, a cultural shift in the medical profession seems necessary. Unless the stigma associated with emotions is removed, the image of the ‘caring doctor’ will remain at the periphery of medical profession, making it difficult for doctors to develop empathy.

The ability to understand and join into someone else’s feelings, fears and concerns presupposes acknowledgement and engagement with one’s own feelings and reactions. Halpern in her work on clinical empathy emphasises the importance of physicians’ awareness of their own feelings and ability to use them to better comprehend “patients’ illness experiences, health habits, psychological needs, and social situations” [2]. According to Halpern, empathy involves “curiosity about others, sensitivity to […] own emotional reactions, and an ongoing capacity to see the patient’s situations, motives, and reactions as distinct from their own”. She suggests that health care professionals who feel “caught emotionally ‘in the morass of the patient’s problems’” [2] should critically reflect on these feelings and unpick their triggers and origins. Self-reflection is an important instrument for avoiding burnout as well as for developing empathy. This process can be facilitated by providing tools such as questionnaires that prompt physicians to review and think about their experiences and the emotions they provoke [37].

Self-reflection is not the only method physicians can use when dealing with their emotions. Sharing experiences with colleagues, friends and family could also encourage and support physicians to reflect on their feelings. Particularly, the external view from colleagues or supervisors could facilitate the emotional labour required [38]. Various forms of support and discussion groups for health care professionals have been developed since the 1950s, when Balint groups, where general practitioners could discuss and share emotionally difficult experiences with their peers, were first introduced in London [39]. More recent techniques include mindfulness courses for medical students and physicians [40–42]; narrative medicine [1] or Schwartz rounds, where staff come together to discuss the non-clinical aspects of caring for patients in a multidisciplinary group [3, 43]. Yet, despite the variety of stress management techniques, evidence show that physicians rarely make use of the support available [44, 45]. This is not only due to the stigma associated with revealing emotions but also to a lack of time in clinical practice [46, 47]. Long working hours and heavy work load are not conducive to mental wellbeing [36]. A stressed and overworked doctor rarely has the possibility or the mental resources to participate in Balint groups, or attend mindfulness courses. Under such conditions, ignoring or hiding feelings seems as the easiest way out. It is not only the problem of the availability of remedial tools that needs to be addressed. Of equal importance is the creation of appropriate working conditions that would allow physicians to make use of these tools and integrate them into their professional routine.

## Conclusions

Empathy is an important aspect of clinical care, but the emotional labour it requires is not negligible. The ability for empathy, namely the joining into someone else’s feelings and emotions, presupposes a high level of self-awareness of one’s own emotions. Physicians are routinely trained to remain detached from their own as well as their patients’ emotions, perpetuating the ideal of the skilled and cool-minded professional. They have to deal daily with distressing situations, heavy workload, and strict time constrains. Such an environment is not conducive to the development of empathy in the contact with patients. Although a certain degree of detachment is important for doctors, over-detachment characterised by emotional neglect and denial can have serious consequences for both physicians and patients. A variety of support groups and courses are already available for doctors to help them develop emotional self-awareness and self-reflection, but only few make use of them. Until the stigma of the ‘unprofessional’ and ‘weak’ doctor who needs support is removed and the work conditions improve, these tools will remain unutilised. Empathy should not only be expected from doctors but should be actively promoted, assisted and cultivated in the medical profession.

## Footnotes

1 We acknowledge that empathy is a fundamental requirement in all healthcare professions. However, in this paper we focus only on physicians, and on how the professional ideal of the ‘rational’ and ‘detached’ doctor can inhibit the development of empathy. We believe that this problem is particularly pronounced in the case of doctors, where the expression of emotions is more likely to be seen as ‘unprofessional’. Although not all medical specialties (e.g. GPs vs surgeons) are exposed to the same types of emotional encounters with patients, we maintain that empathy remains a basic skill of all types of medical practice.
